# Hautkrebsfrüherkennung in der alternden Bevölkerung Sachsen-Anhalts: Inanspruchnahme sowie förderliche und hinderliche Faktoren

**DOI:** 10.1007/s00105-023-05238-y

**Published:** 2023-10-23

**Authors:** S. Walter, C. Hasenpusch, I. Hrudey, J. Holstiege, J. Bätzing, H. Faßhauer, S. March, E. Swart, C. Stallmann

**Affiliations:** 1 Institut für Sozialmedizin und Gesundheitssystemforschung, Medizinische Fakultät, Otto-von-Guericke-Universität, Leipziger Str. 44, 39120 Magdeburg, Deutschland; 2 Fachbereich Soziale Arbeit, Gesundheit und Medien, Hochschule Magdeburg-Stendal, Magdeburg, Deutschland; 3 Fachbereich Epidemiologie und Versorgungsatlas, Zentralinstitut für die Kassenärztliche Versorgung in Deutschland, Berlin, Deutschland

**Keywords:** Krebsfrüherkennungsprogramm, Screening, Prävention, Melanom, Ältere Bevölkerung, Cancer screening program, Melanoma, Cancer screening program, Prevention, Older population

## Abstract

**Hintergrund:**

Das gesetzliche Hautkrebsscreening (gHKS) kann einem schweren Krankheitsverlauf von verschiedenen Hautkrebsarten vorbeugen. Das Beispiel des malignen Melanoms zeigt, dass es angesichts des hohen durchschnittlichen Erkrankungsalters von 62 Jahren (Frauen) und 68 Jahren (Männer) für die alternde Bevölkerung bedeutsam ist. Für Sachsen-Anhalt (ST) als ein vom demografischen Wandel besonders betroffenes Land gibt es für das gHKS wenige Daten zur ausführlichen Abbildung der Nutzung.

**Ziel:**

Für die Studie werden die Teilnahmeraten des gHKS für Personen ab 55 Jahren in ST im Vergleich zum restlichen Bundesgebiet dargestellt. Gründe und Barrieren der Teilnahme aus Sicht der Inanspruchnahmeberechtigten sowie mögliche Handlungsfelder werden aufgezeigt.

**Material und Methoden:**

Für die Zielgruppe werden deutschlandweite ambulante vertragsärztliche Abrechnungsdaten zum gHKS von 2011 bis 2020 quer- und längsschnittlich analysiert. Leitfadengestützte Telefoninterviews mit 18 Einwohner*innen dienen unter Anwendung der qualitativen Inhaltsanalyse nach Kuckartz der Identifikation von Gründen und Barrieren der Inanspruchnahme des gHKS.

**Ergebnisse:**

Das gHKS wurde in ST und dem restlichen Bundesgebiet 2011 bis 2020 selten und unregelmäßig in Anspruch genommen – die jährliche Inanspruchnahmerate lag jeweils ca. bei 8,0 % (Bundesdurchschnitt: 8,4 %). Etwa 50 % der anspruchsberechtigten Personen ab 55 Jahren nahmen zwischen 2011 und 2020 gar nicht am gHKS teil. Die höchsten Inanspruchnahmeraten hatten Männer zwischen 70 und 79 Jahren. Neben kreisspezifischen Unterschieden, die auf mögliche Versorgungslücken hindeuten, scheinen insbesondere Informationsdefizite die geringe Teilnahme zu bedingen.

**Diskussion:**

Die geringe Inanspruchnahme des gHKS u. a. aufgrund von Informationsdefiziten unter den Anspruchsberechtigten erfordert zielgruppenspezifische Informationsangebote.

## Abstract (en)

**Background:**

Statutory skin cancer screening (gHKS) can counteract severe courses of various types of skin cancer. The example of malignant melanoma shows that screening is important for older adults in view of the average age of onset of this disease: 62 (women) and 68 years (men). For Saxony–Anhalt (ST), as a state particularly affected by demographic change, little gHKS data are available regarding its usage.

**Objectives:**

For the study, gHKS participation rates for persons aged 55 and older are presented for ST in comparison to the rest of Germany. Reasons for and barriers to participation from those eligible as well as possible fields of action are shown.

**Materials and methods:**

For the target group, outpatient billing data from the Central Institute for Statutory Health Insurance Physicians in Germany (Zi) on gHKS from 2011–2020 are cross-sectionally and longitudinally analysed. Guideline-based telephone interviews with 18 residents using qualitative content analysis according to Kuckartz serve to identify reasons for and barriers to the use of gHKS.

**Results:**

The gHKS was rarely and irregularly used in ST and other federal states of Germany from 2011–2020; the annual utilisation rate was about 8.0% (national average: 8.4%). Between 2011 and 2020, 50% of eligible people aged 55 years and older did not participate in the gHKS. The highest utilisation rates were among men aged 70–79 years. In addition to district-specific differences, which indicate possible gaps in care, information deficits appear to be the main reason for low participation.

**Conclusion:**

The low utilisation of gHKS, partly due to a lack of information among those entitled to it, requires target group-specific information services.

## Hintergrund

Bösartige Neubildungen der Haut gehören zu den häufigsten Krebsarten in Deutschland [1, 2]. Bei den vorrangig vorkommende Hautkrebsformen wird zwischen malignen Melanomen (schwarzer Hautkrebs) und nichtmelanotischen Melanomen (heller Hautkrebs) – wie das Basalzellkarzinom und das Plattenepithelkarzinom – unterschieden [2]. Das maligne Melanom stellt aufgrund der hohen Wahrscheinlichkeit der Metastasenbildung die bösartigste Tumorform dar [3]. Im Jahr 2018 lag die Inzidenzrate des malignen Melanoms in Deutschland je 100.000 Personen bei 18,9 (Frauen) bzw. 20,2 (Männer). Geschlechtsspezifische Differenzen zeigen sich im mittleren Erkrankungsalter, bei 62 (Frauen) bzw. 68 Jahren (Männer) [2]. Die hellen Hautkrebsformen treten im Vergleich zum schwarzen Hautkrebs häufiger auf [2]. Die Inzidenzrate für die nichtmelanotischen Hautkrebserkrankungen betrug 2018 bei Frauen 122,4 und bei den Männern 152,2 je 100.000 Personen [2]; 75 % der hellen Hautkrebsform sind Basalzellkarzinome und 25 % Plattenepithelkarzinome [2]. Männer erkranken an hellem Hautkrebs häufiger als Frauen [2]. Sowohl bei dem schwarzen als auch bei dem hellen Hautkrebs ist die Erkrankungshäufigkeit altersabhängig: Das Erkrankungsrisiko steigt mit zunehmendem Alter, die meisten Hautkrebserkrankungen werden zwischen 75 und 79 Jahren diagnostiziert [3, 4].

Maligne Veränderungen der Haut und deren Vorstufen sollen im Rahmen des Hautkrebsscreenings frühzeitig erkannt werden, um die Heilungschancen durch eine frühzeitig eingeleitete Therapie zu erhöhen und weitere Morbidität zu verhindern [3, 5]. Aufgrund der hohen Prävalenz nahm der Gemeinsame Bundesausschuss das gesetzliche Hautkrebsscreening (gHKS) zum 01.07.2008 als präventive Regelleistung in den Abrechnungskatalog der gesetzlichen Krankenversicherung (GKV) auf [6]. Seitdem besteht für gesetzlich Versicherte ab 35 Jahren alle 2 Jahre ein kostenfreier Anspruch auf eine standardisierte Ganzkörperinspektion der Haut durch Dermatolog*innen oder Allgemeinmediziner*innen mit entsprechender Zusatzqualifikation, wobei Letztere bundesweit bevorzugt zur Leistungserbringung aufgesucht werden [7].

Mit 27,6 % hat Sachsen-Anhalt (ST) aktuell den im Bundesvergleich höchsten Anteil an über 65-jährigen Einwohner*innen (EW) [8] – Tendenz steigend. Eine regelmäßige, informierte Inanspruchnahme des gHKS scheint trotz mangelnder Erkenntnisse zur Mortalitätssenkung [7] in Anbetracht des mit dem Alter steigenden Risikos für eine Hautkrebserkrankung für die alternde Bevölkerung in ST relevant. Bisher liegen jedoch kaum Erkenntnisse zur jährlichen sowie zur regelmäßigen Inanspruchnahme des gHKS für ST vor. Mit diesem Beitrag werden neben den Inanspruchnahmeraten für ST im Bundesvergleich auch förderliche und hinderliche Faktoren, die bei Personen ab 55 Jahren in ST zur (Nicht‑)Teilnahme am gHKS führen, analysiert und in abschließenden Empfehlungen für eine gesteigerte informierte Inanspruchnahme diskutiert.

## Methodik

Die Inanspruchnahme von Leistungen der Primär- und Sekundärprävention, unter anderem des gHKS, wurde in der vom Europäischen Fonds für regionale Entwicklung (EFRE) und dem Land Sachsen-Anhalt geförderten Studie „Prävention im Alter Sachsen-Anhalt“ (PrimA LSA) multimethodisch exploriert. Als Teilprojekt des Forschungsverbundes „Autonomie im Alter“ (AiA) wurden durch das Institut für Sozialmedizin und Gesundheitssystemforschung der Medizinischen Fakultät der Otto-von-Guericke-Universität Magdeburg und den Fachbereich Soziale Arbeit, Gesundheit und Medien der Hochschule Magdeburg-Stendal (Vorhabennummern: ZS/2019/07/99610, ZS/2020/06/145442) die Inanspruchnahme und Determinanten von GKV-finanzierten Präventionsleistungen bei Menschen ab 55 Jahren in ST untersucht mit dem Ziel, Ansätze für die Steigerung der (informierten) Inanspruchnahme abzuleiten. Das gesamte Studiendesign ist an anderer Stelle detailliert publiziert [9].

Im vorliegenden Beitrag wird die Inanspruchnahme des gHKS mittels kassenübergreifender, vertragsärztlicher Abrechnungsdaten entsprechend dem Einheitlichen Bewertungsmaßstab (EBM) gemäß § 295 Sozialgesetzbuch V (SGB V) im Beobachtungszeitraum 2011 bis 2020 quer- und längsschnittlich analysiert. Die Daten liegen aggregiert nach Geschlecht und Altersklassen für ST und dessen 14 Landkreise und kreisfreie Städte (LK) sowie als Vergleichswerte für das restliche Bundesgebiet insgesamt sowie gesondert für Berlin, die restlichen östlichen (ÖBL, ohne ST) und westlichen Bundesländer (WBL, ohne Bayern und Baden-Württemberg) vor. Die direkte Alters- und Geschlechtsstandardisierung erfolgte unter Verwendung der Alters- und Geschlechtsstruktur aller gesetzlich Krankenversicherten des Jahres 2011.

Für die längsschnittliche Analyse wurde eine Kohorte aus Versicherten ab 50 Jahren gebildet, die seit 2011 durchgängig in den vertragsärztlichen Abrechnungsdaten beobachtet werden und bis einschließlich 2020 jährlich (beliebige) vertragsärztliche Leistungen in Anspruch genommen hatten. Insgesamt umfasste die Studienpopulation für die querschnittliche Analyse im Jahr 2020 für ST 932.335 Personen (N_Bund ohne ST_ = 18.802.525). Die längsschnittlichen Inanspruchnahmeraten am gHKS wurden anhand der Regelmäßigkeit der Teilnahme über 10 Jahre wie folgt ausgewiesen:

- regelmäßige, turnusgemäße Inanspruchnahme (5-mal in 10 Jahren als maximale Anspruchsberechtigung),
- unregelmäßige Inanspruchnahme (1- bis 4‑mal in 10 Jahren),
- Nicht-Inanspruchnahme (0-mal in 10 Jahren).

Als Einschlusskriterien für die Inanspruchnahme am gHKS galt die vertragsärztliche Abrechnung der EBM-Nr. 01745.

Die Inanspruchnahmeraten werden durch qualitativ erhobene subjektive Patientenperspektiven ergänzt. Dazu wurden leitfadengestützte Telefoninterviews mit Sachsen-Anhalter*innen ab 55 Jahren geführt. Studienteilnehmer*innen aus je 2 ländlich und städtisch geprägten Regionen in ST wurden über beigelegte Flyer einer schriftlichen Befragung sowie durch die direkte Ansprache von Kontakten des Studienteams rekrutiert [9]. Insgesamt wurden 18 Personen zu ihrem Wissen, der Einstellung und der Inanspruchnahme verschiedener präventiver Regelleistungen sowie zu förderlichen und hinderlichen Faktoren bezüglich der Teilnahme befragt. In Tab. 1 sind die Charakteristika der Interviewteilnehmenden ersichtlich.

**Table 1:** 

Merkmal/Kriterium	Ausprägung	*N*	(%)
Geschlecht	Männlich	7	39
	Weiblich	11	61
Region	Magdeburg	8	44
	Halle (Saale)	4	22
	LK Mansfeld-Südharz	2	11
	LK Börde	4	22
Alter (in Jahren)	Durchschnitt	62	–
Teilnahme gHKS	Bereits mindestens 1‑mal	13	72
	Davon männlich	5	28

Die Telefoninterviews wurden als Audiodateien aufgezeichnet und wortwörtlich transkribiert. Die qualitative Inhaltsanalyse erfolgte softwaregestützt mit MAXQDA (VERBI Software GmbH, Berlin) nach Kuckartz [10]. Auf Grundlage des Interviewleitfadens wurden zunächst deduktiv Haupt- und Unterkategorien abgeleitet, und ein Kodierleitfaden wurde erstellt. Weitere (Unter‑)Kategorienbildung erfolgte induktiv entsprechend dem Interviewmaterial. Insgesamt wurden 9 Haupt- und 18 Unterkategorien gebildet. Zwei Personen kodierten die Interviews anhand des stetig erweiterten Kodierleitfadens unabhängig voneinander. Die Kodierungen wurden anschließend verglichen, bei Diskrepanzen mit einer weiteren Person diskutiert und basierend auf der konsensuellen Methode angeglichen [11].

## Ergebnisse

**Figure 1:**
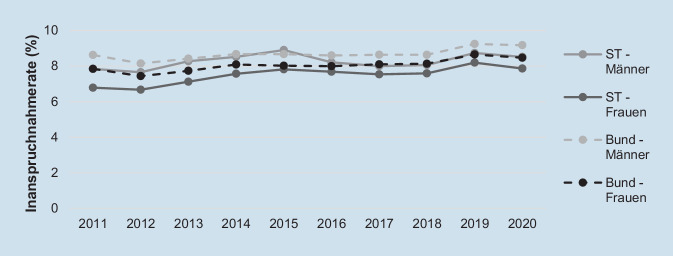

Seit 2011 wurde das gHKS jährlich in ST und im übrigen Bundesgebiet kontinuierlich von etwa 8,0 % der Anspruchsberechtigten ab 55 Jahren in Anspruch genommen. Dabei wurde das gHKS zwischen 2011 und 2020 im übrigen Bundesgebiet ohne ST von Personen ab 55 Jahren etwas häufiger in Anspruch genommen (im Mittel 8,4 %) als in ST (im Mittel 7,9 %) – ausgenommen dem Jahr 2015, in dem die Teilnahme am gHKS bei Männern in ST über dem Bundesdurchschnitt lag (Abb. 1). Über alle Jahre hinweg nahmen im übrigen Bundesgebiet und in ST insgesamt mit etwa 1 bis 2 Prozentpunkten mehr Männer als Frauen ab 55 Jahren am gHKS teil, wobei sich die Inanspruchnahmeraten beider Geschlechter ab 2016 etwas annäherten.

Frauen zwischen 55 und 69 Jahren in ST und dem übrigen Bundesgebiet nahmen das gHKS 2011 bis 2020 öfter in Anspruch als die gleichaltrigen Männer. Ab 70 Jahren wurde das gHKS dagegen öfter durch Männer als durch gleichaltrige Frauen sowohl in ST als auch dem übrigen Bundesgebiet in Anspruch genommen (Tab. 2). Die Inanspruchnahmerate des gHKS stieg bei Frauen und Männern zwischen 55 und 69 Jahren an. Die höchsten Werte werden über den gesamten Untersuchungszeitraum 2011 bis 2020 bei den 70- bis 79-jährigen Frauen und Männern beobachtet. Ab etwa 80 Jahren nehmen in ST und dem übrigen Bundesgebiet erneut weniger Frauen und Männer am gHKS teil.

**Table 2:** 

		55–59		60–64		65–69		70–74		75–79		80–84		85–89		90–94		95+	
Jahr	–	Männer	Frauen	Männer	Frauen	Männer	Frauen	Männer	Frauen	Männer	Frauen	Männer	Frauen	Männer	Frauen	Männer	Frauen	Männer	Frauen
2011	ST	6,5	8,0	7,7	9,3	10,1	10,4	10,2	9,6	9,3	7,8	7,9	6,0	6,5	4,6	4,7	3,2	–	2,2
	Bund	7,8	9,2	9,1	10,3	10,9	11,4	11,6	10,7	10,9	9,1	9,3	7,1	7,9	5,5	6,1	4,2	3,9	3,1
2012	ST	6,3	7,8	7,6	8,7	9,3	9,8	9,8	9,1	9,0	7,6	7,7	5,8	6,4	4,7	5,0	3,4	–	*3,1*
	Bund	7,4	8,8	8,6	9,7	10,2	10,7	10,9	10,3	10,4	8,8	8,8	6,7	7,4	5,2	5,7	3,9	3,9	*2,9*
2013	ST	6,7	8,0	7,7	9,3	9,9	10,6	10,9	10,3	10,1	8,8	8,2	6,4	6,8	4,8	*5,9*	3,5	–	2,3
	Bund	7,6	9,1	8,8	10,0	10,3	11,0	11,5	10,9	11,1	9,4	9,3	7,0	7,7	5,4	*5,7*	3,9	3,6	2,9
2014	ST	7,0	8,6	8,1	9,8	10,2	11,0	11,4	10,9	10,6	9,0	8,7	6,7	7,0	5,2	5,2	4,0	–	*2,9*
	Bund	7,8	9,6	9,0	10,3	10,5	11,3	11,8	11,6	11,5	9,9	9,7	7,5	8,0	5,6	6,1	4,1	3,6	*2,9*
2015	ST	7,0	8,8	8,3	9,7	10,0	*11,2*	*11,7*	11,5	11,5	*10,0*	9,6	7,5	7,6	5,2	5,6	3,7	–	*2,9*
	Bund	7,7	9,4	8,7	10,1	10,2	*11,1*	*11,7*	11,6	11,7	*10,0*	10,0	7,6	8,0	5,5	6,0	3,9	3,9	*2,9*
2016	ST	6,8	8,4	7,8	9,3	*10,0*	10,8	11,2	11,3	11,0	9,8	9,3	7,3	7,0	5,3	*6,1*	3,7	*4,7*	*3,2*
	Bund	7,5	9,4	8,5	9,9	*10,0*	11,0	11,3	11,4	11,6	10,2	10,1	7,7	7,9	5,5	*6,0*	4,0	*4,3*	*2,9*
2017	ST	6,5	8,3	7,7	8,9	9,2	10,5	11,0	10,8	11,4	9,9	9,7	7,4	7,4	5,2	5,5	3,7	3,6	*3,0*
	Bund	7,5	9,4	8,4	10,0	10,0	11,0	11,3	11,5	11,8	10,5	10,5	8,0	8,1	5,5	6,0	3,9	4,1	*2,9*
2018	ST	6,6	8,3	7,5	9,0	9,4	10,4	10,8	11,1	11,5	10,0	9,9	7,7	7,6	5,2	5,6	3,7	3,7	*2,9*
	Bund	7,5	9,6	8,3	9,9	10,0	11,0	11,0	11,3	11,8	10,7	10,7	8,2	8,2	5,6	6,2	3,9	4,0	*2,9*
2019	ST	7,1	9,2	8,0	9,5	10,0	11,1	11,5	11,4	12,2	11,1	10,7	8,4	8,2	5,7	6,1	3,9	*4,8*	*3,4*
	Bund	8,2	10,2	9,0	10,6	10,6	11,7	11,6	12,0	12,6	11,5	11,4	8,9	8,8	6,0	6,5	4,1	*4,5*	*2,9*
2020	ST	7,2	8,7	8,0	8,9	9,3	10,4	10,9	10,8	12,0	10,9	10,8	8,4	8,2	6,0	5,7	4,0	4,3	2,6
	Bund	8,4	9,9	9,1	10,2	10,4	11,1	11,3	11,4	12,2	11,1	11,3	8,9	9,0	6,3	6,4	4,2	4,5	3,1

^a^Höhere Raten in ST im Vergleich zum Bund hervorgehoben

**Figure 2:**
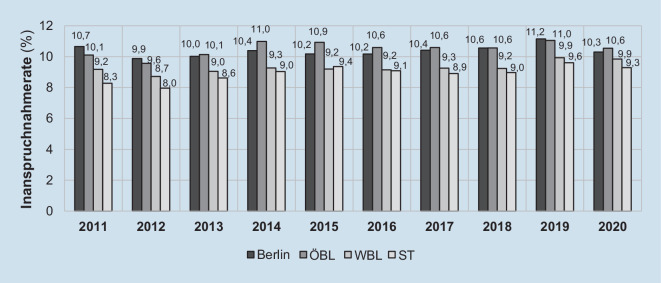

Auch im regionalen Vergleich wurde das gHKS von Personen ab 55 Jahren in ST 2011 bis 2020 seltener in Anspruch genommen. Die Inanspruchnahme für ST (Mittelwert [MW]_2011–2020_ = 8,9 %) ist deutlich niedriger als in Berlin (MW_2011–2020_ = 10,4 %), den übrigen ÖBL (MW_2011–2020_ = 10,5 %) und den WBL (MW_2011–2020_ = 9,3 %), wobei sich seit 2015 die Raten an Letztere annähern (Abb. 2).

**Figure 3:**
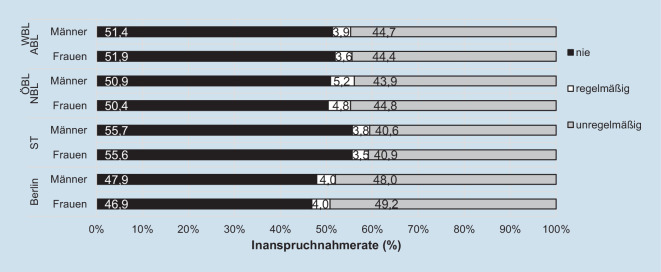

Zwischen 2011 und 2020 hätten die Anspruchsberechtigten turnusgemäß 5‑mal am gHKS teilnehmen können. Dem kamen v. a. Männer (5,2 %) und Frauen (4,8 %) aus den restlichen ÖBL nach. In ST nahmen lediglich 3,5 % der Frauen und 3,8 % der Männer ab 55 Jahren regelmäßig am gHKS teil – was jeweils mit den Werten der WBL vergleichbar ist (Abb. 3). In ST, den restlichen ÖBL und den WBL überwiegt der Anteil an Personen, die zwischen 2011 und 2020 gar nicht am gHKS teilnahmen (Range: 50,4 % [Frauen in den ÖBL] – 55,7 % [Männer in ST]), deutlich gegenüber der unregelmäßigen Inanspruchnahme. Dagegen ist – mit geringerem Unterschied – die Rate der unregelmäßigen Inanspruchnahme in Berlin etwas höher als die der Nicht-Teilnahme zwischen 2011 und 2020. In ST liegen die Nicht-Inanspruchnahmeraten bei Männern (55,7 %) und Frauen (55,6 %) zwischen 2011 und 2020 deutlich über den unregelmäßigen Inanspruchnahmeraten (Männer: 40,6 %; Frauen: 40,9 %). Damit war ST zwischen 2011 und 2020 die Region mit der niedrigsten regelmäßigen Teilnahme und gleichzeitig der höchsten Nicht-Teilnahme am gHKS. Im Vergleich der Regelmäßigkeit der Inanspruchnahme zeigen sich zwischen 2011 und 2020 bundesweit auch in ST kaum Unterschiede zwischen Männern und Frauen.

Auch im 10-Jahres-Längsschnitt zeigt sich, dass insbesondere Personen zwischen 60 und 79 Jahren regelmäßig am gHKS teilnahmen. Dabei war bei Personen dieser Altersklasse aus ST und den ABL die niedrigste regelmäßige Teilnahme (4,6 %) zu verzeichnen – und eine damit deutlich geringere als bei gleichaltrigen Personen aus Berlin (5,1 %) und den übrigen ÖBL (6,4 %). In ST lag über 10 Jahre die Nicht-Teilnahme am gHKS insbesondere in der Altersgruppe von 55 bis 59 Jahren (58,7 %) und in der Altersgruppe über 80 Jahren (63,5 %) deutlich über den Nicht-Inanspruchnahmeraten der übrigen ÖBL und Berlins.

**Figure 4:**
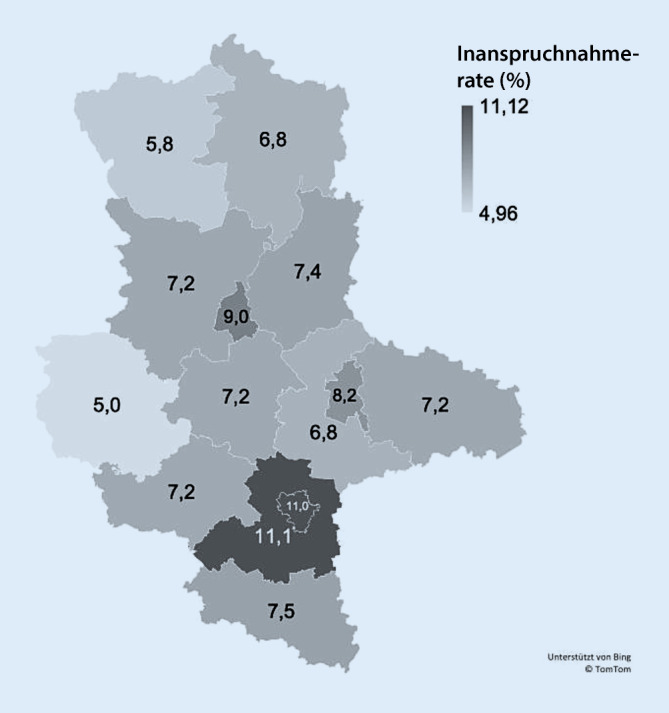

Das gHKS wurde zwischen 2011 und 2020 in ST v. a. durch Personen ab 55 Jahren aus dem LK Saalekreis (MW_2011–2020_ = 11,1 %) und Halle/Saale (MW_2011–2020_ = 11,0 %) – Süd-ST – in Anspruch genommen. Zwischen 2011 und 2020 nahmen zudem im Mittel 9,0 % der Anspruchsberechtigten in der Landeshauptstadt Magdeburg am gHKS teil (Abb. 4). In diesen Regionen lag die Inanspruchnahme über dem Bundesdurchschnitt (MW_2011–2020_ = 8,4 %).

Über die Jahre war die Inanspruchnahme des gHKS auch in den verschiedenen Landkreisen relativ konstant (Tab. 3), wobei in der Mehrheit bis 2015/2016 ein leichter Anstieg zu verzeichnen war. Dieser Trend setzte sich bis 2020 nicht fort: 2016 bis 2020 war die Inanspruchnahme in der Mehrheit der LK eher rückläufig. Mit Hinblick auf die COVID-19-Pandemie zeigen sich 2020 in der Mehrheit der LK leichte Rückgänge der Teilnahme am gHKS, jedoch auch erhöhte Inanspruchnahmeraten in den LK Anhalt-Bitterfeld (+0,1 %), Harz (+0,1 %), Jerichower Land (+0,5 %), Stendal (+0,9 %) und Magdeburg (+1,0 %) im Vergleich zu 2019. Die geringsten jährlichen Inanspruchnahmen wiesen durchgängig der Altmarkkreis Salzwedel (MW_2011–2020_ = 5,8 %) und der LK Harz (MW_2011–2020_ = 5,0 %) auf. Sie liegen deutlich unter dem Bundesdurchschnitt (s. oben).

**Table 3:** 

Jahr	Bund	Altmarkkreis Salzwedel	LK Anhalt-Bitterfeld	LK Börde	Burgenlandkreis	Dessau-Roßlau	Halle/Saale	LK Harz	LK Jerichower Land	Magdeburg	LK Mansfeld-Südharz	Saalekreis	Salzlandkreis	LK Stendal	LK Wittenberg
2011	8,2	6,1	6,7	6,8	6,8	7,3	*9,9*	4,2	6,5	*8,2*	6,9	*11,0*	5,9	6,9	6,8
2012	7,8	6,2	6,0	7,2	6,7	7,6	*9,7*	3,8	5,7	*8,0*	6,4	*10,6*	5,7	7,0	6,9
2013	8,1	5,5	6,8	6,8	7,3	*8,2*	*10,3*	4,5	7,0	*8,7*	6,8	*10,8*	6,9	7,1	6,7
2014	8,4	6,5	7,0	7,5	7,7	8,0	*10,5*	4,7	6,4	*9,3*	7,0	*11,4*	7,6	7,3	7,8
2015	8,3	6,0	6,7	7,5	8,0	8,1	*11,1*	5,2	*9,1*	*9,1*	7,1	*11,0*	7,7	7,7	7,1
2016	8,3	5,7	6,4	7,4	7,8	8,1	*11,6*	5,3	7,6	*8,7*	7,1	*11,5*	7,4	6,9	7,9
2017	8,4	5,3	7,0	7,7	7,5	*9,8*	*11,6*	5,1	*8,6*	*8,0*	7,1	*11,1*	7,7	5,0	7,3
2018	8,4	5,4	6,6	7,1	7,1	7,9	*11,6*	5,3	7,6	*9,0*	7,5	*11,5*	7,8	5,9	8,3
2019	9,0	5,8	7,4	7,6	8,3	8,7	*12,3*	5,6	7,8	*10,1*	8,0	*11,6*	8,2	6,4	7,5
2020	8,9	5,5	7,5	7,0	7,8	8,3	*11,8*	5,9	8,3	*11,0*	8,0	*10,7*	7,2	7,3	6,4

**Figure 5:**
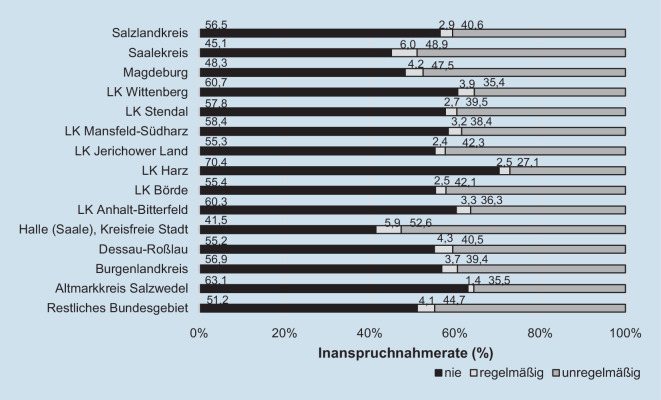

Bundesweit haben zwischen 2011 und 2020 51,2 % der Anspruchsberechtigten ab 55 Jahren nicht am gHKS teilgenommen (Abb. 5). Ausgenommen Halle/Saale, Magdeburg und dem Saalekreis liegen alle anderen LK ST bei der Nicht-Teilnahmerate über diesem Bundesdurchschnitt, wobei der LK Harz mit 70,4 % nicht-teilnehmenden Anspruchsberechtigten hierbei deutlich abweicht.

### Gründe und Barrieren für Personen ab 55 Jahren

Die Mehrheit der insgesamt 18 Interviewteilnehmenden (*n* = 13) nahm nach eigenen Angaben bereits am gHKS teil, wobei hier die Häufigkeit und Regelmäßigkeit der Teilnahme nicht spezifiziert wurden. Die Personen ab 55 Jahren nahmen vorwiegend in allgemeinmedizinischen Praxen am gHKS teil und wurden aufgrund der regelmäßigen allgemeinmedizinischen Versorgung niedrigschwellig zur Teilnahme am gHKS veranlasst. Chronische Erkrankungen wie Diabetes mellitus führten zur turnusgemäßen Vorstellung in der Praxis und zur medizinischen Kontrolle. Die behandelnden Allgemeinmediziner*innen nutzten nach den Interviewaussagen diese Gelegenheiten, um das gHKS direkt zu erbringen, daran zu erinnern oder konkrete Empfehlungen zur Teilnahme auszusprechen, z. B. in Form von Überweisungen. Diese Behandlungskombinationen suggerierten einigen Interviewteilnehmenden, dass das gHKS Teil der allgemeinen Gesundheitsuntersuchung („Gesundheits-Check-up“ [GU]) sei und eine Teilnahme am gHKS entsprechend keines zusätzlichen Aufwands bedürfe. Eine Interviewteilnehmerin berichtete, dass sie den GU alle 2 Jahre durch den Hausarzt wahrnimmt: „Ähm, […], weil da […] ja auch, glaube ich, ein Hautcheck mit dabei [ist]“ (weiblich, 59 Jahre). Weitere Interviewteilnehmende schilderten, das gHKS aufgrund individueller Risikofaktoren regelmäßig gezielt in Anspruch zu nehmen. Eine subjektiv wahrgenommene empfindliche Haut sowie Leberflecke als potenzielle Risikofaktoren und/oder häufiger Aufenthalt im Freien stellen dabei die häufigsten Gründe dar. Darüber hinaus veranlassten Erkrankungsfälle im sozialen Umfeld sowie vergangene, eigene Haut(krebs)erkrankungen zur regelmäßigen Teilnahme am gHKS. Diese Motive basieren insbesondere auf einem Sicherheitsbedürfnis, das zur regelmäßige Überprüfung der eigenen Gesundheit führt.

Die 5 Interviewteilnehmenden, die das gHKS bisher nicht in Anspruch genommen haben, begründeten dies v. a. mit mangelndem Wissen über die Leistung(serbringung). Mehrere Interviewteilnehmenden äußerten, dass bisher keine dermatologische Inanspruchnahme stattfand und der Zugang zu Hautärzt*innen damit subjektiv nicht gegeben sei: „[Einen] Hautarzt, […] habe ich […] keinen festen hier, weil ich […] noch keine Probleme hatte. Da habe ich […] kaum Möglichkeiten, dass man […] drangenommen wird […]. Und viele nehmen überhaupt keine neuen Patienten“ (männlich, 62 Jahre). Resümierend gaben die Nicht-Teilnehmenden an, keine Beschwerden zu haben, sodass sich die Notwendigkeit des gHKS erübrige.

## Diskussion

Das gHKS wurde in den vergangenen Jahren bundesweit von Personen ab 55 Jahren vergleichsweise selten – und v. a. unregelmäßig – in Anspruch genommen. Die turnusgemäße Inanspruchnahme war sehr gering und lag bundesweit und in ST unter vergleichbaren Ergebnissen bei AOK-Versicherten [12]. Ausgenommen in Halle/Saale, dem LK Saalekreis und der Landeshauptstadt Magdeburg lag die Inanspruchnahme in ST deutlich unter dem Bundesdurchschnitt.

Bei altersspezifischer Betrachtung zeigt sich sowohl für ST als auch das übrige Bundesgebiet, dass mehr Frauen im Alter zwischen 55 und 69 Jahren am gHKS teilnahmen als gleichaltrige Männer; dieses Verhältnis kehrt sich im höheren Alter um. Diese Erkenntnis deckt sich mit Ergebnissen anderer Studien [12, 13]. Die höchste Inanspruchnahme zeigte sich bei Personen zwischen 70 und 79 Jahren und korreliert mit dem Gipfel diagnostizierter Hautkrebserkrankungen [3, 4]. Entsprechend sollten insbesondere jüngere, teils noch berufstätige Männer und Rentnerinnen Zielgruppen für Maßnahmen zur Steigerung der Inanspruchnahme des gHKS sein.

Entsprechend dem Evaluationsbericht zum gHKS [7] nahmen auch die befragten Sachsen-Anhalter*innen das gHKS überwiegend bei Allgemeinmediziner*innen mit entsprechender Zusatzqualifikation in Anspruch. Oftmals erfolgte dies im Rahmen regulärer Kontrolluntersuchungen oder der GU, sodass eine kombinierte Leistungserbringung erfolgen konnte. Jedoch ist seit der Erhöhung des GU-Intervalls auf 3 Jahre, eine Kombination in einem längeren Turnus von lediglich 6 Jahren möglich. Des Weiteren ist anzunehmen, dass die Unterlassung von aufschiebbaren Untersuchungen zur Entlastung des Gesundheitssystems aufgrund der COVID-19-Pandemie anteilig mit einem Rückgang der Inanspruchnahmeraten 2020 verbunden ist [12, 14].

Nach Angaben der Interviewteilnehmenden setzte die kombinierte Inanspruchnahme des gHKS das aktive Zutun der Ärzt*innen voraus. Die Befragten agierten nach eigenen Aussagen in der Mehrheit eher passiv und leisteten Empfehlungen Folge. Dies unterstreicht einerseits die Relevanz niedrigschwelliger Angebote sowie die zentrale Funktion der Allgemeinmediziner*innen als sog. „Gatekeeper“. In ihrer Rolle können sie Versicherte sensibilisieren, Ansprüche kommunizieren und die Leistung entweder direkt erbringen oder durch Überweisungen vermitteln. Möglicherweise ist diese spezielle Funktion ein weiterer Grund dafür, dass das gHKS vorwiegend durch Allgemeinmediziner*innen erbracht wurde. Die Wissensvermittlung scheint von zentraler Bedeutung: Einige Interviewteilnehmende gaben an, nicht gewusst zu haben, dass für sie ein regelmäßiger Anspruch auf das gHKS bestehe. Eissing et al. [15] kommen zu dem Ergebnis, dass lediglich die Hälfte der Anspruchsberechtigten um ihren Anspruch auf das gHKS wüsste; wobei dieses Wissen mit 56,0 % Inanspruchnahme des gHKS einhergehe. Bestehende Informationsdefizite werden ebenfalls innerhalb der Interviewaussagen deutlich. Durch die subjektive Wahrnehmung, „gesund“ und frei von Beschwerden zu sein, erscheint die Teilnahme am gHKS nicht nötig. Diese Einstellung und damit einhergehende Nicht-Inanspruchnahme reduzieren das Potenzial des gHKS. Informationsdefizite seitens der Versicherten sind damit zentrale Barrieren der Inanspruchnahme des gHKS [5, 13].

Dass die Befragten das gHKS insbesondere in Hausarztpraxen in Anspruch nehmen, könnte neben der direkten Ansprache in ST auch mit der vergleichsweise geringen Dermatolog*innendichte zusammenhängen. Im Jahr 2022 waren 4,0 Dermatolog*innen je 100.000 EW in ST niedergelassen und damit weniger als z. B. in Berlin (6,3/100.000 EW) [16]. Im Vergleich zu Mecklenburg-Vorpommern (MVP), einem Bundesland mit einer ähnlichen Verteilung der Raumordnungskategorien, zeigt sich, dass, obwohl die hautärztliche Dichte (4,1/100.000 EW) vergleichbar mit ST ist, dort eine um 2 Prozentpunkte höhere Inanspruchnahme unter Mitgliedern und Versicherten der GKV vorliegt (10,7 %) [16, 17]. Ein möglicher Grund könnte die höhere Dichte an hausärztlichen Praxen in MVP (74,6/100.000 EW vs. ST: 65,4/100.000 EW) sein, die potenziell das gHKS bei entsprechender Qualifizierung durchführen können [16]. In ST zeigt sich zudem ein leichtes Nord-Süd-Gefälle bezüglich der Dermatolog*innendichte: Während in Nord-ST auf 100.000 EW 1,0–3,3 Dermatolog*innen kommen, sind es in Süd-ST bereits 5,5 [16]. Dies könnte auch erklären, warum die Inanspruchnahmeraten insbesondere in Halle/Saale und dem umliegenden Saalekreis (Süd-ST), also im stärker verdichteten Süden des Bundeslandes, höher ausfallen und über dem Bundesdurchschnitt liegen. Es müssen damit auch strukturelle Unterschiede innerhalb des Landes beachtet werden. Wie auch von einem Interviewteilnehmenden angemerkt, folgt daraus, dass insbesondere in Nord-ST der Besuch einer dermatologischen Praxis entweder gar nicht oder nur mit sehr langer Wartezeit auf einen Termin möglich ist, was Einfluss auf die (regelmäßige) Inanspruchnahme des gHKS haben könnte. Insbesondere auch (altersbedingte) Immobilität könnte hier ein weiterer negativer Einflussfaktor sein, da für dermatologische Versorgung oft größere Entfernungen zurückgelegt werden müssen. In diesem Zusammenhang sind ggf. auch die Förderung telemedizinischer dermatologischer Leistungen und die präventionsdermatologische Fortbildung in der Allgemeinmedizin zu entwickeln und zu stärken.

## Fazit für die Praxis

- (Regelmäßige) Inanspruchnahme des Hautkrebsscreenings seit Einführung 2008 konstant gering.
- Inanspruchnahmeraten in Sachsen-Anhalt (ausgenommen Süd-Sachsen-Anhalt und Magdeburg) geringer als im bundesweiten Vergleich.
- Vor allem mangelndes Wissen um Untersuchung(srhythmus) und mangelnder Zugang zu dermatologischen Praxen als Barrieren bei Personen ab 55 Jahren in Sachsen-Anhalt.
- Informationsdefizite hindern Versicherte, selbstbestimmt zu agieren, und erfordern aktives Zutun von (Allgemein‑)Mediziner*innen – dieses setzt Kapazitäten in den Praxen voraus, welche nur bedingt gegeben sind.
- Gezieltere, zielgruppenspezifische, niedrigschwellige Informationen notwendig, z. B. als organisiertes Programm, öffentliche Kampagnen und vermehrte Hinweise beispielsweise durch die Krankenversicherung oder andere Akteure.
