# Publisher Erratum zu: Hautkrebsfrüherkennung in der alternden Bevölkerung Sachsen-Anhalts

**DOI:** 10.1007/s00105-023-05263-x

**Published:** 2023-11-20

**Authors:** S. Walter, C. Hasenpusch, I. Hrudey, J. Holstiege, J. Bätzing, H. Faßhauer, S. March, E. Swart, C. Stallmann

**Affiliations:** 1 Institut für Sozialmedizin und Gesundheitssystemforschung, Medizinische Fakultät, Otto-von-Guericke-Universität, Leipziger Str. 44, 39120 Magdeburg, Deutschland; 2 Fachbereich Soziale Arbeit, Gesundheit und Medien, Hochschule Magdeburg-Stendal, Magdeburg, Deutschland; 3 Fachbereich Epidemiologie und Versorgungsatlas, Zentralinstitut für die Kassenärztliche Versorgung in Deutschland, Berlin, Deutschland

**Erratum zu:**

**Dermatologie 2023**

10.1007/s00105-023-05238-y

In diesem Artikel wurde der Name der Autorin I. Hrudey fälschlicherweise als H. Hrudey angegeben. Der Originalartikel wurde korrigiert.

